# Artificial Intelligence for Automatic Measurement of Sagittal Vertical Axis Using ResUNet Framework

**DOI:** 10.3390/jcm8111826

**Published:** 2019-11-01

**Authors:** Chi-Hung Weng, Chih-Li Wang, Yu-Jui Huang, Yu-Cheng Yeh, Chen-Ju Fu, Chao-Yuan Yeh, Tsung-Ting Tsai

**Affiliations:** 1 aetherAI Co., Ltd., Taipei 115, Taiwan; chihung@aetherai.com; 2 Department of Orthopaedic Surgery, Spine Division, Bone and Joint Research Center, Chang Gung Memorial Hospital and Chang Gung University College of Medicine, Taoyuan 333, Taiwan; alexwang7508@gmail.com (C.-L.W.); rr821028@gmail.com (Y.-J.H.); yehchrist2@gmail.com (Y.-C.Y.); 3 Department of Medical Imaging and Intervention, Chang Gung Memorial Hospital and Chang Gung University College of Medicine, Taoyuan 333, Taiwan; chenjufu@gmail.com

**Keywords:** artificial intelligence, convolutional neural network, resunet, sagittal vertical axis

## Abstract

We present an automated method for measuring the sagittal vertical axis (SVA) from lateral radiography of whole spine using a convolutional neural network for keypoint detection (ResUNet) with our improved localization method. The algorithm is robust to various clinical conditions, such as degenerative changes or deformities. The ResUNet was trained and evaluated on 990 standing lateral radiographs taken at Chang Gung Memorial Hospital, Linkou and performs SVA measurement with median absolute error of 1.183 ± 0.166 mm. The 5-mm detection rate of the C7 body and the sacrum are 91% and 87%, respectively. The SVA calculation takes approximately 0.2 s per image. The intra-class correlation coefficient of the SVA estimates between the algorithm and physicians of different years of experience ranges from 0.946 to 0.993, indicating an excellent consistency. The superior performance of the proposed method and its high consistency with physicians proved its usefulness for automatic measurement of SVA in clinical settings.

## 1. Introduction

Adult spinal deformity (ASD) is a broad diagnosis referring to stable asymptomatic curves and disabling deformities in the spine that contribute to pain, weakness, and low health-related quality of life (HRQOL). Although ASD is quite common, the variation and unique pattern of each spinal deformity make reproducible measurement difficult. Previous studies investigating the correlation between radiographic appearances and clinical symptoms yielded rather low predictive power due to highly variable health status [1]. Little correlation between radiographic assessment and questionnaire scores was found for adolescent idiopathic scoliosis [2]. However, in 2005, Glassman et al. showed that sagittal plane balance is the most reliable predictor of clinical symptoms in adults with spinal deformity [3]. Even mild positive sagittal balance results in destructive spinal deformity, clinical symptoms of which deteriorate linearly [4]. Therefore, a surgical plan for restoring sagittal balance is crucial in all spinal reconstructive surgeries [3]. 

Parameters to describe the sagittal alignment of the spine include the sagittal vertical axis (SVA), thoracic kyphosis, lumbar lordosis, pelvic incidence, pelvic tilt, and sacral slope. SVA, which is the most commonly used measure of the sagittal plane, is obtained from standing lateral radiographs and is defined as the horizontal distance between two landmarks: the center of C7 vertebral body and the posterior superior aspect of the S1 vertebral body. According to Schwab’s classification based on Jean Dubousset‘s cone of economy, the realignment of the spine should aim to meet the criteria of SVA < 50 mm to alleviate sensations of falling over [5]. Consequently, formulating a patient-specific surgical plan requires SVA measurement both preoperatively and postoperatively, using whole-spine lateral radiographs. However, manual SVA estimation on radiographs is rather inconvenient due to lack of easy-to-use tools. To solve this issue, we propose using deep-learning models for fully automatic estimation of SVA.

A well-known family of deep-learning models is the convolutional neural networks (CNNs). CNNs have drawn considerable attention since 2012, as they were found to outperform traditional image processing algorithms on image classification tasks [6]. Since then, CNNs have been increasingly used for medical image analysis [7,8]. A CNN is primarily made of stacked convolution layers, which can be regarded as a series of learnable feature extractors designed for acquiring low-to-high-level features of an image. In general, basic image features, such as blob of colors or edges of different orientations, are learnable by the shallow convolution layers. On the other hand, complex image features, such as appearances of objects, are learnable by the deep convolution layers [9]. Therefore, well-trained convolution layers can be used to extract informative features that are useful for landmark localization or other specific tasks [10,11].

Automatic SVA estimation can generally be regarded as the task of landmark localization, as SVA was defined as the horizontal difference between two anatomical landmarks. In this study, we investigated the performance of ResUNet [12] for automatic vertebrae localization on radiographs. ResUNet is a variant of CNN. Its UNet-like structure [13] combines the contextual information produced by deep layers and better location information produced by shallow layers, allowing better utilization of low- and high-level features. In addition, the encapsulated residual blocks [14] of ResUNet enable better flow of information and can avoid performance deterioration as the network goes deeper. ResUNet has been used for cell segmentation from biomedical images [12] and road extraction from satellite images [15]. To the best of our knowledge, our study is the first to utilize ResUNet for anatomical landmark localization. 

Some previous works use CNN for vertebrae segmentation or localization. For example, for 3D CT and MR images, Lessmann et al. [16] used a 3D UNet-like model for vertebrae segmentation. In addition, Wimmer et al. [17] used 3D CNN for vertebrae localization. There were also studies on radiographs: for biplanar radiographs, including A-P and lateral view, Gallbusera et al. [18] used a database collected using the EOS™ imaging system [19] and trained CNN models for each of the landmarks. For lateral spine radiographs, Al Arif et al. [20] applied a UNet model for the localization of cervical vertebral centers. 

In this study, we applied ResUNet on plain lateral spine radiographs. Although our algorithm is similar to the one in [20], there are some major differences. First, we did not split images into patches, as patching leads to extra pre- and post-processing steps, and the correlation between landmarks of different patches will be ignored completely. However, if patching was not performed, the large image size would lead to insufficient amount of RAM on GPU. To alleviate this issue, we used a small batch size (batch size = 2) and replaced the widely used batch normalization with the group normalization [21] in our network. Group normalization is known to perform well even when the batch size is small, as it does not perform normalization along the batch axis. Secondly, we let probability maps of landmarks to be predicted separately. Thus, predictions were not on the same map and further post-processing steps involving separation and identification of the landmarks can be eliminated.

Our main contribution in this paper is three-fold. Firstly, we validated that automatic SVA estimation on lateral spine radiography is feasible using the current deep-learning techniques. The procedure of 10-fold cross-validation was applied to validate the effectiveness of the constructed SVA estimator (ResUNet). Secondly, modern networks often take the Gaussian heatmaps centering at landmark locations as the targets of regression [22,23,24]. In this study, we experimented also the use of exponential heatmaps for model selection. Our experiments suggested that, when the heatmap function was narrow, the exponential heatmaps can be more easily learned, as compared with the Gaussian heatmaps. Thirdly, we compared the estimates between ResUNet and human experts of different seniority. The results show that they were in excellent agreement.

## 2. Materials and Methods

### 2.1. Data Preparation

We collected 990 whole spine lateral radiographs from 990 patients who had degenerative changes or deformities in the spine from January 2012 to September 2018 at Chang Gung Memorial Hospital, Linkou, Taiwan. The collected radiographs were from different X-ray scanners and the mean pixel resolution was 3552×1434. Poor-quality images such as those with poor image contrast and positioning errors were excluded. Among the collected radiographs, 13% (128) of the images are of children (age < 12), 42% (414) of the images are of young adults (age 13–18) and 45% (448) of the images are of adults (age > 18). Overall, 77% (765) of the images do not have an implant, while 23% (225) do. In the group without implants (765), 56% (428) of the patients have scoliosis, while 44% (337) of patients have degenerative change. In the group with implants (229), 76% (170) of the implants extend from thoracic to lumbar, 21% (48) of the implants extend from lumbar to sacral, 2% (5) of the implants only involve lumbar, and 1% (2) of the implants only involve thoracic. Pediatric and young adult patients diagnosed of scoliosis were enrolled in this study. In terms of sacral morphology, this cohort displayed various degrees of physiological sacral fusions, as well as pathology resulting from scoliosis. The images were anonymized, and standard research protocols were followed. This study was approved by the Institutional Ethical Committee (IRB number: 201801651B0 (1809270049)).

**Figure 1:**
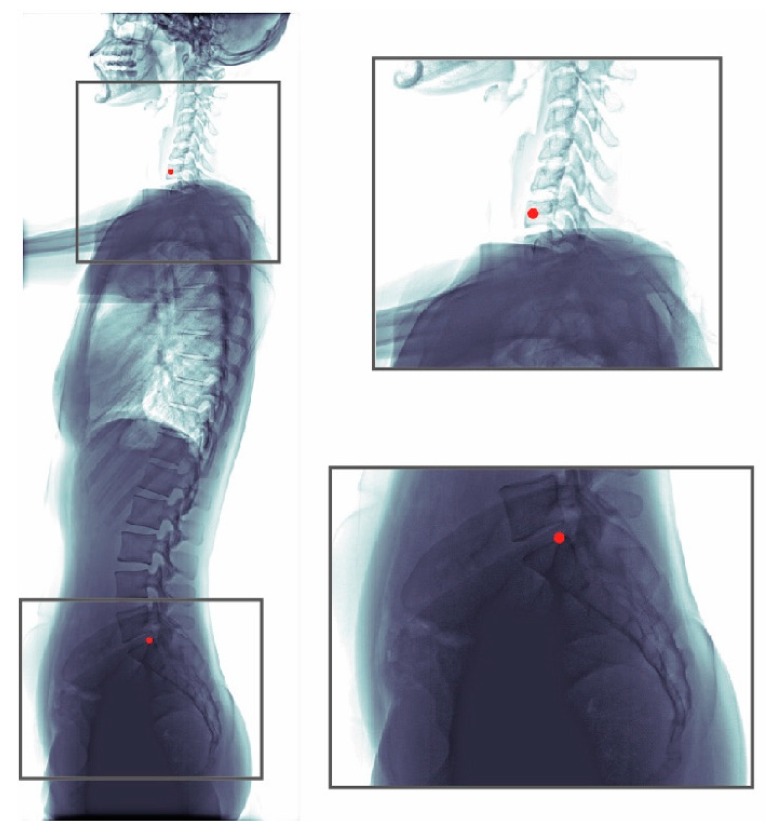
Annotations of a radiograph. The C7 center and the posterior corner of the sacrum were annotated (red dots).

The whole dataset (990 images) was annotated by one senior resident orthopedic surgeon. This surgeon has more than five years of experience for manual measurement of spine parameters. When a difficult case was encountered, the surgeon discussed with an experienced spine surgeon to reach a consensus. This annotated dataset was used for model training and evaluation. An annotated radiograph can be seen in Figure 1. We used a custom written MATLAB GUI program for annotation.

In addition, an experienced radiologist and a junior resident physiciane also provided their annotations for a subset of the dataset (99 images), which were randomly selected from the 990 images. Hence, three human annotations (senior/junior resident orthopedic surgeon, and radiologist) can be found for this subset of data and the consistency between our model and human experts can be examined further.

### 2.2. Gaussian and Exponential Heatmaps

A Gaussian heatmap centering at $\left(x_{k},y_{k}\right)$ can be defined as:

(1) $$G_{k}\left(x_{i},y_{j};\;\sigma \right)\;={\;\mathrm{ce}}^{-\frac{{\left(x_{i}-x_{k}\right)}^{2}\;+\;{\left(y_{i}-y_{k}\right)}^{2}}{2\sigma^{2}}},$$

where $\left(x_{i},y_{j}\right)$ is a pixel location of an image (on i-th column and j-th row), $\left(x_{k},y_{k}\right)$ is the human-annotated landmark location, σ is a hyperparameter that controls the width of the Gaussian, and c is the scaling constant, which was fixed to be a large constant (at the order of $10^{3}$) so that the heatmap pixels close to landmark locations were more salient and easier to learn.

Representing landmark locations using heatmaps is beneficial. First, the regression of a heatmap was considered as an easier task as compared to the regression of x and y values, as the latter requires the learning of a highly non-linear function that maps all image pixels into two values only. Secondly, after normalization, the predicted heatmap is more informative, as it can be interpreted as a predicted probability map for the location of a landmark.

However, to determine $G_{k}\left(x_{i},y_{j};\;\sigma \right)$, we need to select σ manually. The value of σ is often set as a small value because wide Gaussians may introduce high uncertainties to the prediction of landmark locations. However, if the Gaussian is not wide enough, features which are useful for prediction but outside the narrow region of Gaussian may have the chance to be ignored, as we simply let the model to predict zeros at the locations of those features.

**Figure 2:**
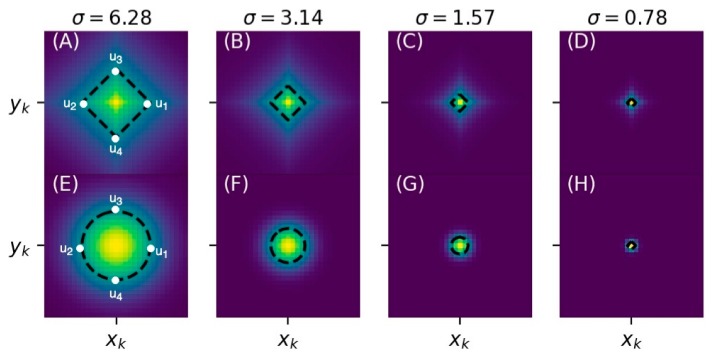
(**A**–**D**) Exponential heatmaps at various σ values; and (**E**–**H**) Gaussian heatmaps at various σ values. We put $\left(x_{k},y_{k}\right)$ at the heatmap center and draw the half-maximum contour of the heatmap (black-dashed lines) for each heatmap plot. When σ is fixed, the half-maxima contours of Gaussian and exponential heatmaps coincide at $u_{1}\;=\;\left(x_{k}+\sigma \sqrt{2\log2},y_{k}\right)$, $u_{2}\;=\;\left(x_{k}\;-\sigma \sqrt{2\log2},y_{k}\right)$, $u_{3}\;=\;\left(x_{k},{\;y}_{k}+\sigma \sqrt{2\log2}\right)$ and $u_{4}\;=\;\left(x_{k},y_{k}-\sigma \sqrt{2\log2}\right)$, as highlighted in (**A**,**E**).

To overcome this issue, we propose the use of exponential heatmaps for landmark localization. An exponential heatmap $E_{k}\left(x_{i},y_{j};\;\alpha \right)$ centering at $\left(x_{k},y_{k}\right)$ can be defined as:

(2) $$E_{k}\left(x_{i},y_{j};\;\alpha \right)\;={\;\mathrm{ce}}^{-\alpha \left(\left|x_{i}-x_{k}\right|+\left|y_{j}-y_{k}\right|\right)},$$

where $\left(x_{k},y_{k}\right)$ is the human-annotated landmark location, α is a hyperparameter that controls the decay rate of the exponential function, and c is the scaling constant as defined in the Gaussian heatmap. For the purpose of comparison, we let $\alpha =\;\frac{1}{\sigma }\sqrt{\log2/2}$ so that both $G_{k}\left(x_{i},y_{j};\;\sigma \right)$ and $E_{k}\left(x_{i},y_{j};\;\sigma \right)$ reach to the same half maxima at $\left(x_{k}\;\pm \sigma \sqrt{2\log2},y_{k}\right)$ and $\left(x_{k},{\;y}_{k}\pm \sigma \sqrt{2\log2}\right)$, as shown in Figure 2.

Compared to the Gaussian heatmap $G_{k}\left(x_{i},y_{j};\;\sigma \right)$, the proposed exponential heatmap $E_{k}\left(x_{i},y_{j};\;\sigma \right)$ has a sharper peak centering at the landmark location. As a result, the predicted locations of landmarks can be less uncertain. Furthermore, $E_{k}\left(x_{i},y_{j};\;\sigma \right)$ decays more slowly (especially when the pixels are close to $y\;={\;y}_{k}$ and $x\;={\;x}_{k}$), which leads to a wider region of heatmap pixels that should be predicted nonzero. In this case, useful features that lie within the non-zero zone are easier to detect and can be used as a clue for easier localization of landmarks.

### 2.3. Model Architecture

**Figure 3:**
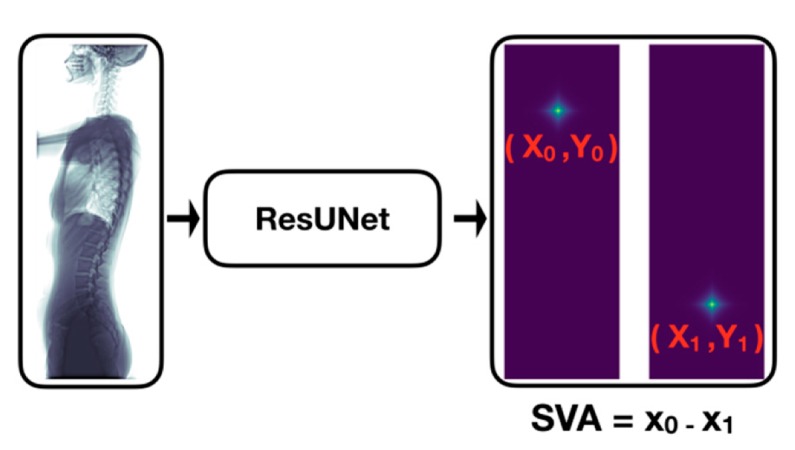
Automatic sagittal vertical axis (SVA) estimator. This estimator will turn an input image (tenor shape = 768×448×1) into predictions of two probability maps of landmarks (tensor shape = 768×448×2 ), which correspond to the C7 center and the posterior corner of the sacrum. The two landmarks are then localized from their corresponding maps and their horizontal difference (i.e., SVA) is calculated.

**Figure 4:**
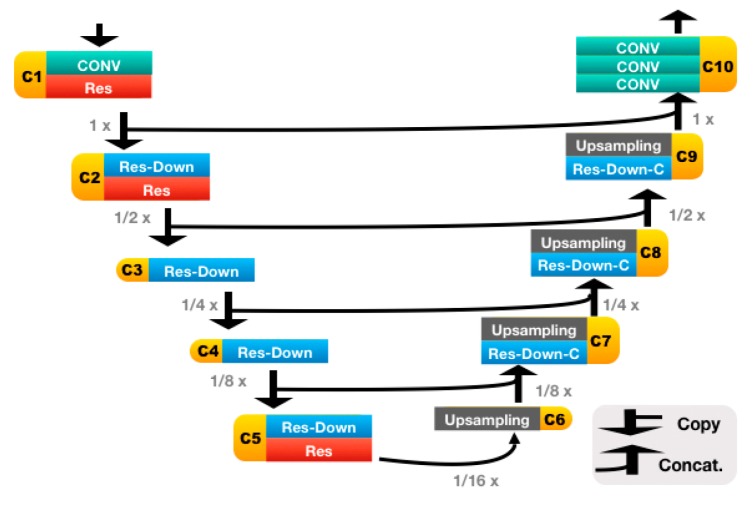
The ResUNet architecture. This network has 31 convolutional layers and is made of the following blocks: CONV, Res, Res-Down, Res-Down-C and Upsampling. The upsampling block performs bilinear upsampling and is non-learnable. The remaining four learnable blocks are illustrated in Figure 5.

Our automatic SVA estimator (Figure 3) was based on a 31-layer ResUNet (Figure 4), which was composed of the head convolutional layers (C1), the downsampling path (C2–C5), upsampling path (C6–C9), and the tail convolutional layers (C10). In the downsampling path, both height and width of the feature maps were reduced by half four times. This process was then reversed during the upsampling path, and two heatmaps of landmarks were predicted immediately after C10.

**Figure 5:**
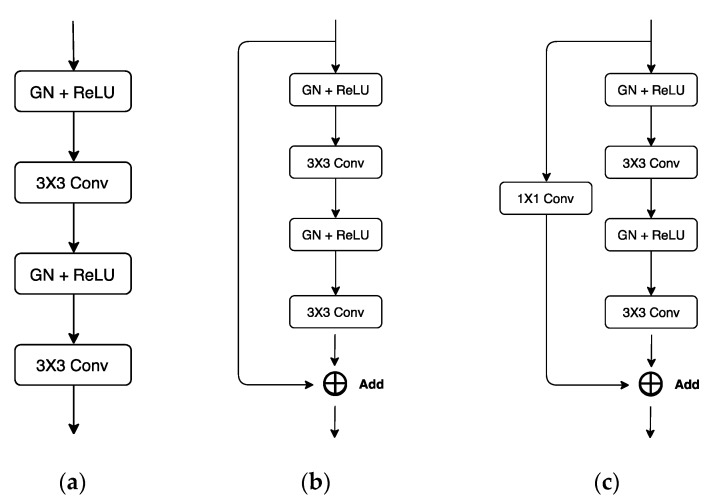
Building blocks of the ResUNet: (**a**) CONV; (**b**) Res; (**c**) Res-Down/Res-Down-C. For all subfigures shown above, GN/ReLU stands for Group Normalization/ Rectified Linear Unit. In addition, CONV and Res were both used for feature extraction, where the latter improved information flow further by adding an extra skip-connection. Res-Down/Res-Down-C was used not only for feature extraction but also for data downsampling, where downsampling was achieved in the dimension of width and height (for Res-Down) or channel (for Res-Down-C). We set strides to 1 for the 1×1 Conv and the first 3×3 Conv in Res-Down. For all other convolutional layers, strides were set to 1.

The details of the ResUNet architecture are further illustrated in Figure 5 and Table 1. It is worth mentioning that, if the C7 center and the posterior corner of the sacrum were to be predicted on the same heatmap, then we would have to cope with landmark extraction and identification. For the former, we would have to, e.g., fit a Gaussian mixture model (which is rather complex) and regard the locations of the fitted Gaussian centers as the landmark locations. For the latter, we perhaps would have to assume that the one above is the landmark of C7, and the one below is the landmark of sacrum, as the extracted landmarks are indistinguishable.

In this work, all landmarks were predicted on separate heatmaps, i.e., the landmarks were separated and distinguishable from the very beginning. Thus, all we need to do was fit an exponential or a Gaussian function to each of the heatmaps and then regarded the obtained center of the fitted function as the estimated location of its corresponding landmark.

### 2.4. Image Preprocessing and Augmentation

Before training, all radiographs were downsized to 768×340 and then padded to 768×448. The padding procedure was crucial, as the radiographs were randomly rotated (angle ∈ [−30°, 30°]) during training, and the vertebrae part of the body should not be missing after rotation.

During training, techniques of data augmentation were applied to the radiographs in order to prevent CNNs from adapting to images of certain scales, orientations, and types of noise. The applied techniques were random scaling (scale ∈[0.9,1.1]), random rotation (angle ∈ [−30°, 30°]), and random Gaussian blur (strength ∈ [0, 0.5]).

### 2.5. Details of Experiments

We trained a 31-layer ResUNet for the localization of the C7 center and the posterior corner of the sacrum. For model selection, the experimented hyperparameters for fine-tuning were heatmaps of different types (Gaussian and exponential) and sizes (σ = 3.14, 1.57, 0.78, 0.39). Furthermore, the procedure of 10-fold cross-validation was followed to test the effectiveness of the constructed models. For a schematic description about how the procedure of 10-fold cross-validation was performed, please refer to Figure S1. 

Two GPUs were used for training (NVIDIA Tesla V100), and the batch size was set to 2 per GPU (therefore, the total batch size was 4). For the procedure of group normalization, the number of groups was set to 32. In addition, mean squared error was chosen as our loss function, which was minimized using stochastic gradient descent (SGD) with Nesterov momentum (momentum = 0.9). To eliminate the effect of overfitting, L2 regularization (strength = 10^−5^) was also added to the loss function.

During training, the learning rate was initially set to 0.01 and was then reduced by half on epoch 100. The number of training epochs was set to 120 and the final model was obtained from the checkpoint that performed the best on the validation data after epoch 80. The elapsed time of an experiment of 10-fold cross-validation was approximately 50 h. We used TensorFlow v1.12 and its Estimator API for model construction and training.

## 3. Results 

We used absolute error of SVA and detection rate of landmarks to evaluate model performance. To obtain errors in pixels, we converted the predicted locations of landmarks back to the resolution of the original (unresized and unpadded) image and then calculated the pixel errors using locations of the true and predicted landmarks. The pixel errors were then converted to mm using the scaling factor recorded in each radiograph. We chose to represent errors in mm because scaling factor is different between radiographs and number of pixels would not be a universal standard. 

### 3.1. Median Absolute Error of SVA

The median absolute error (MAE) of SVA is defined as:

(3) $${\mathrm{MAE}}_{\mathrm{SVA}}\;=\;\mathrm{Median}\left(\left|{\mathrm{SVA}}_{\mathrm{true}}^{\left(1\right)}\;-{\;\mathrm{SVA}}_{\mathrm{pred}.}^{\left(1\right)}\left|,\;\right|{\mathrm{SVA}}_{\mathrm{true}}^{\left(2\right)}\;-{\;\mathrm{SVA}}_{\mathrm{pred}.}^{\left(2\right)}\left|,\;...,\;\right|{\mathrm{SVA}}_{\mathrm{true}}^{\left(N\right)}-{\;\mathrm{SVA}}_{\mathrm{pred}.}^{\left(N\right)}\right|\right),$$

where N stands for number of samples. ${\mathrm{SVA}}_{\mathrm{true}}^{\left(i\right)}$ and ${\mathrm{SVA}}_{\mathrm{pred}.}^{\left(i\right)}$ stand for the i-th true and predicted SVA, respectively. 

As MAE is insensitive to outliers, we also report the number of outliers (>10 mm) for absolute errors. The results of various hyperparameter settings are shown in Table 2.

Our results indicated that, when narrow Gaussian heatmaps were used (σ = 1.57, 0.79), the performance of SVA estimator became highly unstable (standard deviation > 10 mm). These results suggest that σ has to be fine-tuned carefully when choosing Gaussian heatmaps as the regressing targets.

To further investigate the quality of landmark localization, we also calculated the detection rate of the C7 center and the posterior corner of the sacrum, respectively. The results are shown in the following section.

### 3.2. Detection Rate of Landmarks

**Figure 6:**
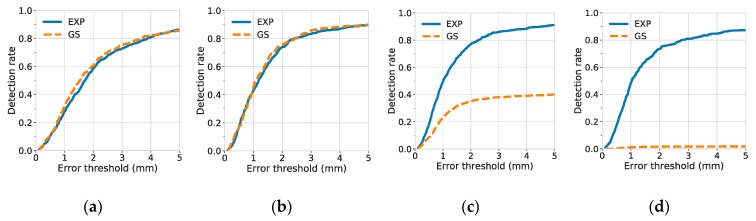
Detection rate of the C7 center against different degrees of error thresholds. The results of various σ values: (**a**) σ = 6.28; (**b**) σ = 3.14; (**c**) σ = 1.57; and (**d**) σ = 0.79. The detection rates were results from 10-fold cross-validation (mean with 1 standard error band). For each figure, EXP and GS stand for exponential and Gaussian heatmaps, respectively.

**Figure 7:**
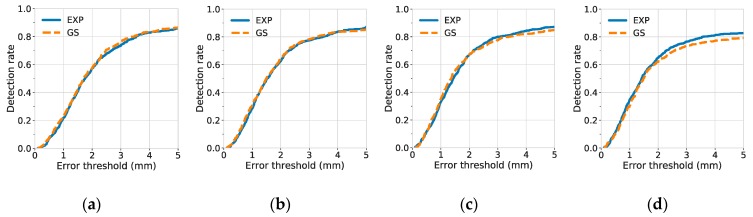
Detection rate of the posterior corner of the sacrum against different degrees of error thresholds. The results of various σ values: (**a**) σ = 6.28; (**b**) σ = 3.14; (**c**) σ = 1.57; and (**d**) σ = 0.79. The detection rates were results from 10-fold cross-validation (mean with 1 standard error band). For each figure, EXP and GS stand for exponential and Gaussian heatmaps, respectively.

A landmark was considered to be detected when the distance between the true and predicted landmark locations was under some acceptable error threshold. To investigate the quality of predicted landmark locations, the detection rate was plotted against different degrees of error thresholds, as shown in Figure 6 (for the C7 center) and Figure 7 (for the posterior corner of the sacrum).

The results of detection rates indicate that the C7 center was difficult to be localized when narrow Gaussian heatmaps were used (σ = 1.57, 0.79). A possible reason is that there were few or no distinct features near the center of the vertebra. The distinct features, such as the vertebrae borders, were not included in the highlighted, non-zero region of the narrow Gaussian and were therefore difficult to be utilized for better localization of landmarks. By contrast, at small σ, the exponential function has not only a narrow region at its center, but also a rather extended region at its tail. Hence, for the exponential function, even if the distinct features were not inside the narrow region, they may still be discovered and utilized, if they were contained within the non-zero, extended tail region.

In contrast to models trained with narrow Gaussian heatmaps, models trained with narrow exponential heatmaps achieved the best performance for the detection of the C7 center. At σ = 1.57, the 1-mm and 5-mm detection rate of the C7 center reached 0.496 ± 0.023 and 0.911 ± 0.012, respectively, as shown in Table 3.

Additionally, we used fixed narrow heatmap function (σ = 1.57) and compared the 5-mm detection rate of models trained with different model complexity (31-layer ResUNet and 12-layer UNet) and different types of heatmaps (Gaussian and exponential). The results are shown in Table 4. Our results suggest that, when the model complexity was fixed, replacing the Gaussian heatmap with the exponential heatmap leads to a better and a more stable performance (the standard deviation has decreased by an order of magnitude). The 5-mm detection rate of C7 increased from 0.045 to 0.842, when a 12-layer UNet was used, and increased from 0.401 to 0.911, when a 31-layer ResUNet was used. These results indicate that the exponential heatmaps were easier to learn at σ = 1.57, regardless of the model complexity. Furthermore, when the heatmap type was fixed, replacing a 12-layer UNet with a 31-layer ResUNet led to significant improvements for the localization of the C7 center. This indicated that the extra skip-connections and convolutional layers of the 31-layer ResUNet were effective at σ = 1.57.

### 3.3. Analysis of Inter-Rater Reliability

To further compare the SVA estimates between ResUNet and human experts, or the SVA estimates between human experts only, we calculated the intra-class correlation coefficient (ICC). With ICC, the agreement between any two estimators can be tested. Our ICC scores were calculated by two-way-random effects model with absolute agreement. For the evaluation of ICC, 99 radiographs from one fold of 10-fold cross-validation were used. 

We report the ICC scores and their 95% confidence intervals in Table 5. The ICC scores were evaluated for four participants, including a 31-layer ResUNet (σ = 1.57, heatmap type = exponential), a junior resident orthopedic surgeon, a senior resident orthopedic surgeon, and a radiologist. Our results show that the ICC scores derived from any pair of the four participants were all >0.9, indicating a high agreement among the four participants.

**Figure 8:**
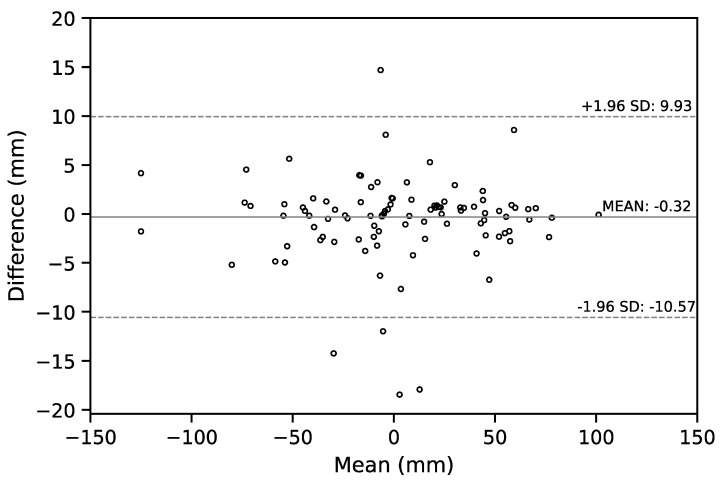
The Bland–Altman plot for the SVA values estimated by the senior resident orthopedic surgeon and the ResUNet model. The horizontal lines of mean difference (solid line) and mean difference ± 1.96 standard deviation (SD) are plotted (dashed lines).

**Figure 9:**
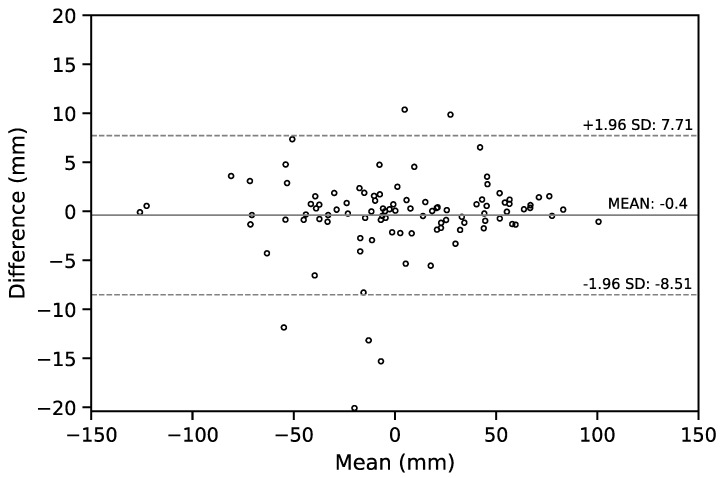
The Bland–Altman plot for the SVA values estimated by the senior and the junior resident orthopedic surgeons. The horizontal lines of mean difference (solid line) and mean difference ± 1.96 standard deviation (SD) are plotted (dashed lines).

We also report the Bland–Altman (B-A) plot for the SVA values estimated by the senior resident orthopedic surgeon and the ResUNet model in Figure 8. The median absolute difference and the mean difference of these two set of estimates was 1.34 mm and −0.32 mm, respectively. For a comparison between doctors, the B-A plot for the SVA values estimated by the senior and the junior resident orthopedic surgeons is shown in Figure 9. The median absolute difference, and the mean difference of these two set of estimates was 1.14 mm and −0.4 mm, respectively.

## 4. Discussion

Sagittal balance is a primary issue for clinical assessment of spine. There are several important morphological indicators of the alignment of spine and pelvis, including sagittal vertical axis (SVA), lumbar lordosis, pelvic incidence, pelvic tilt, and sacral slope. Among them, SVA is the most common parameter used for the evaluation of overall spinal alignment, which represents the sagittal curvature of the spine [25]. SVA can be used to determine the relationship between the spine and pelvis, and has a normal range. Yukawa et al. [26] reported the average value of 3.1 ± 12.6 mm for the SVA of 626 asymptomatic volunteers, and SVA may steadily increase from ages of 30 to 70 years.

Although SVA may change with aging, it remains a reliable sagittal spinopelvic parameter for the assessment of spine alignment. Several authors have demonstrated high inter-rater reliability of SVA measurement by the intra-class correlation coefficient (ICC). Kyrölä et al. [27] reported the ICC score of 0.99 (95% CI: 0.98, 1.00) for SVA in unselected adults with degenerative spine disorders. In a previous study, Katzman et al. [28] also showed a high ICC score of 0.93 (95% CI: 0.83, 0.97) in older adults with hyperkyphosis.

**Figure 10:**
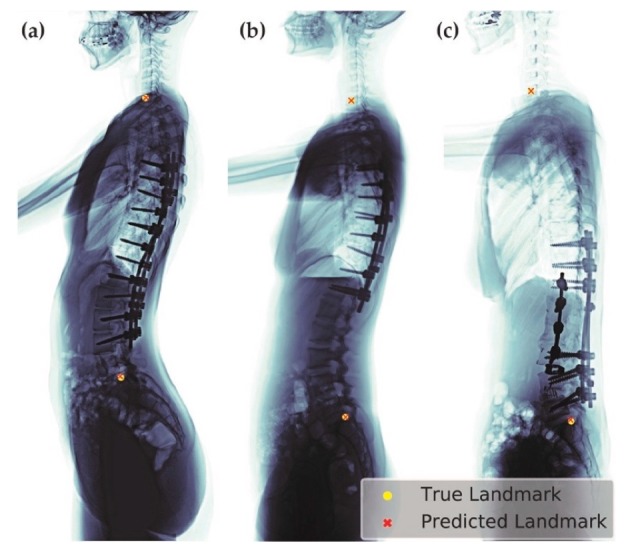
Examples of predicted images of post spinal fusion surgery with varied length of implants: (**a**) thoracic-lumbar fusion; (**b**) thoracic fusion; and (**c**) lumbar-sacral fusion.

**Figure 11:**
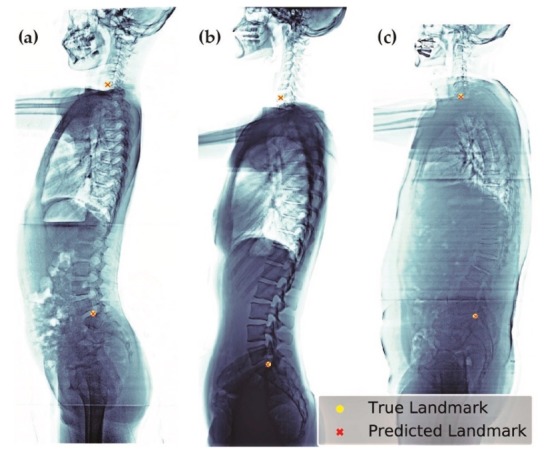
Examples of predicted images of different clinical conditions. These images include patients of: (**a**) ages 8; (**b**) age 23; and (**c**) age 63, who also present with degenerative change.

We selected the best model (σ = 1.57, heatmap type = exponential) based on the absolute error of SVA. This model can be applied to patients of different age groups and with various clinical conditions, including patients with or without implants (Figure 10), having scoliosis, or degenerative changes (Figure 11). 

The hyperparameter, σ, which controls the width of the heatmap function, plays an important role for model selection. In our experiments, we showed that the exponential heatmap allows the model to keep its performance and stability, when σ is small. This is potentially helpful when many landmarks exist. In that situation, fine-tuning σ values for each heatmap is very time-consuming and a heatmap function whose resulting performance is less sensitive to σ may be beneficial.

In this study, the inter-rater reliability of ResUNet and human experts was also examined. We classified the ICC scores similarly to the criteria introduced by Aubin and Kuklo et al. [29,30], i.e. ICC values greater than 0.90 indicate excellent reliability, values between 0.70 and 0.89 indicate good reliability, values between 0.50 and 0.69 indicate moderate reliability, values between 0.25 and 0.49 indicate low reliability, and values less than 0.24 indicate poor reliability. To the best of our knowledge, this study is the first to compare the inter-rater reliability between ResUNet and human experts, and obtained an excellent reliability with the range of ICC values from 0.946 to 0.993.

Using the ResUNet-based framework, the inference time was approximately 0.2 s per image (using one NVIDIA Tesla V100). Due to its short inference time and minimal labor requirement, the automated SVA estimator can be used as an SVA screener for either preoperative or postoperative patients. Nevertheless, recently, abundant important spinal parameters have been proposed and share the same importance as SVA, either at the coronal aspect or lateral aspect. Based on the success of this ResUNet-based SVA estimator, we would like to extend this robust deep learning method to other parameters in the future.

This study has some limitations. Firstly, the outline of vertebrae is slightly torched, as the X-ray was emitted from a single source of radiation. If, for example, the EOS™ X-ray machine [19] can be utilized, then the resulting outline of vertebrae, as well as the landmark locations predicted by our experts, may be more accurate. Secondly, when the selected CV model (σ = 1.57, heatmap type = exponential) was used for predicting the entire dataset (990 images), we found 68 (6.9%) images were with large SVA absolute error (>10 mm). Among these 68 images, there were 33 and 17 images whose predictions were suspected to be influenced by numerical variation of mobile vertebrae (NVMV), and the occlusion of C7 or sacrum, respectively. In the former case, we found it difficult (for both human experts and for ResUNet) to predict the sacral point when confronting with cases of NVMV, or variable sacral fusion, by using the lateral plain radiographs only. NVMV is not uncommon in Chinese adults. In the study of Yan et al. [31], it was shown that 9.6% of patients have the condition of NVMV. We expect our model to handle patients with NVMV better, once more NVMV images are gathered and learned. In the latter case, by using the plain radiography, the C7 vertebral body was sometimes severely occluded by radiopaque areas around the shoulder, e.g., the clavicle, scapula, and humeral head, which may increase the difficulty for the algorithm to learn. By contrast, our human experts were generally able to infer the location of C7 body by observing the context of vertebrae and imagine the alignment of them.

## 5. Conclusions

The automatic measurement of spinal parameters is believed to have a great impact on orthopedics in the coming years. A fully automatic model was developed for the measurement of SVA using a ResUNet framework. The proposed model showed a median absolute error of 1.18 mm. The inference time for one image took approximately 0.2 s, which can be useful for fast screening of large datasets. Our model was cross-validated and fine-tuned to achieve optimal performance with a 31-layer ResUNet. The SVA values estimated by ResUNet was compared with the assessment of experienced doctors and showed excellent agreement. This proposed algorithm can also facilitate the process that allows orthopedic surgeons to design and plan the surgical procedures preoperatively and predict the postoperative outcomes. In addition, this method can be further improved by the ability to detect more landmark points for more clinical application in the future.

## Figures and Tables

**Table 1:** The architecture of a 31-layer ResUNet.

Level	Block	# 3 × 3 Conv	# 1 × 1 Conv	Output Shape (N × H × W × C)
C1	CONV	1	0	N × 768 × 448 × 64
	Res	2	0	N × 768 × 448 × 64
C2	Res-Down	2	1	N × 384 × 224 × 128
	Res	2	0	N × 384 × 224 × 128
C3	Res-Down	2	1	N × 192 × 112 × 256
C4	Res-Down	2	1	N × 96 × 56 × 512
C5	Res-Down	2	1	N × 48 × 28 × 512
	Res	2	0	N × 48 × 28 × 512
C6	Upsampling	0	0	N × 96 × 56 × 512
C7	Res-Down-C	2	1	N × 96 × 56 × 256
	Upsampling	0	0	N × 192 × 112 × 256
C8	Res-Down-C	2	1	N × 192 × 112 × 128
	Upsampling	0	0	N × 384 × 224 × 128
C9	Res-Down-C	2	1	N × 384 × 224 × 64
	Upsampling	0	0	N × 768 × 448 × 64
C10	CONV	1	0	N × 768 × 448 × 64
	CONV	1	0	N × 768 × 448 × 32
	CONV	1	0	N × 768 × 448 × 2

N, Number of images passed into the architecture; H/W/C, Height/Width/Channel of the input images; # 3 × 3 Conv, Number of 3 × 3 convolutional layers; # 1 × 1 Conv, Number of 1 × 1 convolutional layers.

**Table 2:** The median value and number of outliers of the absolute errors of SVA are reported for models constructed using different hyperparameter settings. Mean ± standard deviation (SD) of the median value of the absolute errors of SVA were obtained following the procedure of 10-fold cross-validation.

Heatmap Type	Heatmap Width (σ)	Median Absolute Error of SVA (Mean ± SD, mm)	Number of Outliers (>10 mm)
EXP	6.28	1.570 ± 0.268	67 (6.77%)
GS	6.28	1.508 ± 0.305	54 (5.45%)
EXP	3.14	1.222 ± 0.162	69 (6.97%)
GS	3.14	1.206 ± 0.139	62 (6.26%)
EXP	1.57	1.183 ± 0.166	68 (6.87%)
GS	1.57	9.826 ± 14.1	293 (29.6%)
EXP	0.79	1.210 ± 0.123	82 (8.28%)
GS	0.79	28.80 ± 10.4	588 (59.4%)

EXP, Exponential heatmap; GS, Gaussian heatmap.

**Table 3:** The 1-mm and 5-mm detection rates of models trained using different hyperparameter settings. Mean ± standard deviation (SD) of the detection rates were obtained following the procedure of 10-fold cross-validation.

Heatmap Type	Heatmap Width (σ)	D_C7_ at 1 mm Mean ± SD	D_C7_ at 5 mm	D_S_ at 1 mm	D_S_ at 5 mm
			Mean ± SD	Mean ± SD	Mean ± SD
EXP	6.28	0.279 ± 0.013	0.863 ± 0.015	0.220 ± 0.014	0.859 ± 0.011
GS	6.28	0.313 ± 0.027	0.858 ± 0.011	0.235 ± 0.023	0.867 ± 0.0093
EXP	3.14	0.433 ± 0.017	0.899 ± 0.0046	0.289 ± 0.029	0.872 ± 0.0054
GS	3.14	0.460 ± 0.016	0.898 ± 0.0093	0.309 ± 0.028	0.853 ± 0.015
EXP	1.57	0.496 ± 0.023	0.911 ± 0.012	0.328 ± 0.016	0.871 ± 0.0037
GS	1.57	0.231 ± 0.13	0.401 ± 0.23	0.354 ± 0.018	0.849 ± 0.026
EXP	0.79	0.487 ± 0.026	0.873 ± 0.012	0.349 ± 0.012	0.827 ± 0.016
GS	0.79	0.012 ± 0.050	0.018 ± 0.039	0.307 ± 0.013	0.792 ± 0.026

EXP, Exponential heatmap; GS, Gaussian heatmap; D_C7_, Detection rate of the C7 center; D_S_, Detection rate of the posterior corner of the sacrum.

**Table 4:** The 5-mm detection rates of models trained using σ = 1.57. Mean ± standard deviation (SD) of the detection rates were obtained following the procedure of 10-fold cross-validation.

Model	Number of Layers	Heatmap Type	D_C7_ at 5 mm	D_S_ at 5 mm
			Mean ± SD	Mean ± SD
UNet	12	GS	0.045 ± 0.078	0.750 ± 0.039
UNet	12	EXP	0.842 ± 0.0079	0.853 ± 0.0093
ResUNet	31	GS	0.401 ± 0.23	0.849 ± 0.026
ResUNet	31	EXP	0.911 ± 0.012	0.871 ± 0.0037

EXP, Exponential heatmap; GS, Gaussian heatmap; D_C7_, Detection rate of the C7 center; D_S_, Detection rate of the posterior corner of the sacrum.

**Table 5:** The intra-class correlation coefficient (ICC) and its 95% confidence interval (CI). The ICC values were evaluated for different pair of estimators.

Measurement: SVA (mm)	ICC with 95% CI	95% CI
ResUNet vs. Rater 1 ^1^	0.989	0.984–0.993
ResUNet vs. Rater 2 ^2^	0.946	0.920–0.963
ResUNet vs. Rater 3 ^2^	0.993	0.989–0.995
Rater 1 vs. Rater 2	0.949	0.925–0.966
Rater 1 vs. Rater 3	0.996	0.993–0.997
Rater 2 vs. Rater 3	0.955	0.934–0.970

^1^ Rater 1, Radiologist. ^2^ Rater 2 (Rater 3), Junior (Senior) resident orthopedic surgeon.

## Supplementary Materials

The following are available online at https://www.mdpi.com/2077-0383/8/11/1826/s1, Figure S1: Schematic outline of the procedure of 10-fold cross-validation.
