# Supplementary material for: Artificial Intelligence for Automatic Measurement of Sagittal Vertical Axis Using ResUNet Framework
Source: J Clin Med. 2019 Nov 1;8(11):1826. doi: 10.3390/jcm8111826 (PMC6912675; doi:10.3390/jcm8111826)
Supplement: Supplementary file 1 [file jcm-08-01826-s001.pdf]

Figure S1.

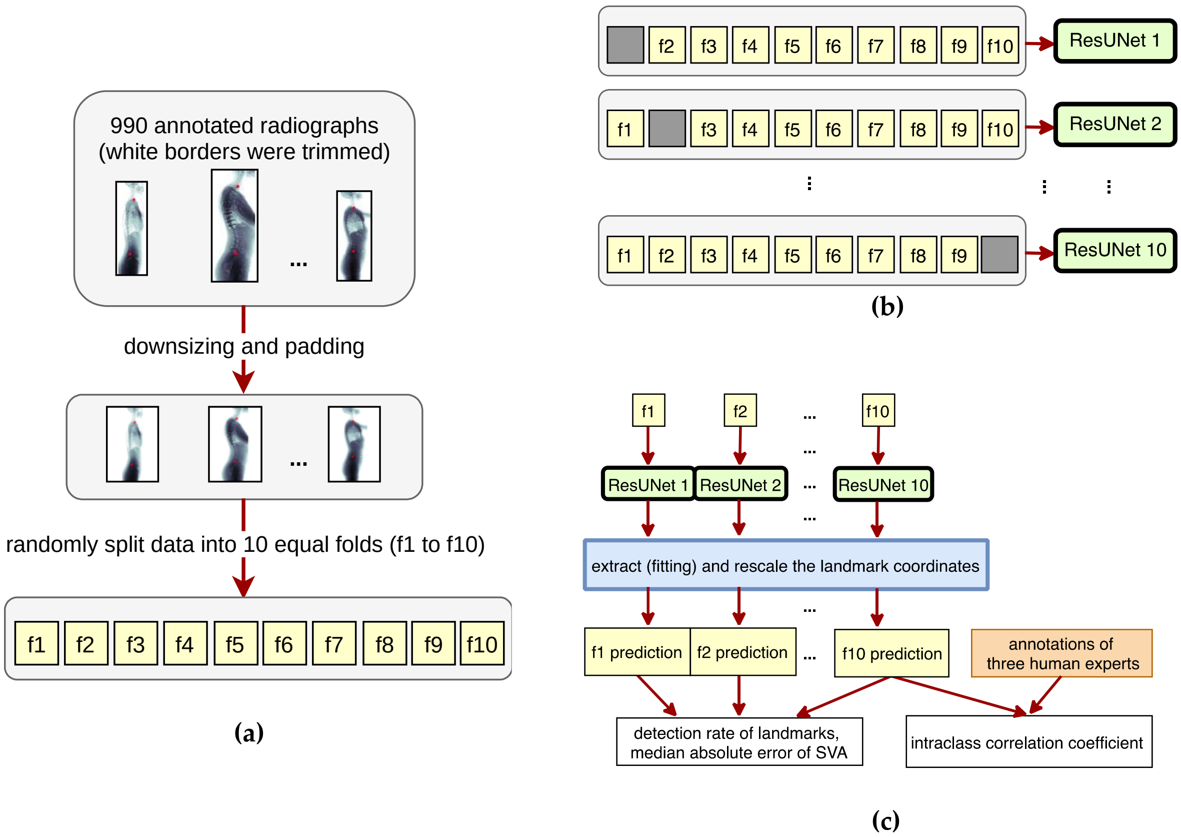

Figure S1. Schematic outline of the procedure of 10-fold cross-validation. The procedure is consisted of three subsequent steps (from a to c): (**a**) data preprocessing for training; (**b**) model training; (**c**) model evaluation.
